# Brain activities responding to acupuncture at acupoint in healthy subjects: a study protocol based on task-based fMRI

**DOI:** 10.3389/fneur.2025.1655478

**Published:** 2025-10-13

**Authors:** Xingying Lu, Juan Ou, Liyao Ding, Wei Dang, Yongfeng Liu, Jinhuan Zhang

**Affiliations:** ^1^Department of Acupuncture, Shenzhen Traditional Chinese Medicine Hospital, Shenzhen, China; ^2^Department of Acupuncture, The Fourth Clinical Medical College of Guangzhou University of Chinese Medicine, Shenzhen, China; ^3^Department of Acupuncture, The Seventh Clinical Medical College of Guangzhou University of Chinese Medicine, Shenzhen, China

**Keywords:** acupuncture, healthy subjects, functional magnetic resonance imaging, randomized controlled trial, protocol

## Abstract

**Background:**

Acupuncture is a widely used complementary therapy; however, the central mechanisms underlying its effects, particularly how stimulation at different specific acupoints modulates brain function in distinct or common ways, remain poorly understood. This gap persists due to a lack of large-sample, systematic comparative studies under a unified experimental paradigm. Task-based and resting-state functional magnetic resonance imaging (fMRI) offer powerful tools to capture both the instant and sustained neural responses to acupuncture.

**Methods:**

We designed a randomized, single-blind, controlled trial. To achieve high statistical power and generalizability, 250 healthy participants will be enrolled. Each participant will undergo acupuncture at one of seven predefined acupoints (verum) and its corresponding non-acupoint (sham control) in two separate sessions, with a 1-week interval. Each session includes: (1) resting-state fMRI before and after needle manipulation, and (2) task-fMRI during the manipulation. The primary outcomes are fMRI-derived brain activity and functional connectivity patterns. Blinding assessment and the Modified Massachusetts General Hospital Acupuncture Sensation Scale-Chinese version (C-MMASS) will be collected to evaluate the credibility of sham control and the Deqi sensation.

**Discussion:**

This study is novel in its comprehensive approach to mapping the neural correlates of multiple acupoints within a single, rigorous design. We anticipate that our results will provide the first systematic characterization of the “acupoint-brain functional network” map, elucidating both common activation patterns across acupoints and acupoint-specific differential responses. This will significantly contribute to understanding the functional neuroanatomy of acupuncture and provide high-level evidence for its mechanism of action, ultimately helping to bridge the gap between traditional practice and modern neuroscience.

**Conclusion:**

The findings of this trial are expected to establish a robust empirical foundation for the neural basis of acupuncture, offering insights that could validate clinical practice and guide future target-specific acupuncture applications.

**Clinical trial registration:**

Identifier ITMCTR2025000066.

## 1 Introduction

Acupuncture, an ancient Chinese therapeutic practice dating back over 2,000 years (1), is a non-pharmacological therapy rooted in Traditional Chinese Medicine (TCM) and its Meridian Theory. It is believed to regulate bodily functions by stimulating vital energy (Qi) at specific acupoints, which then travels along meridians to treat various diseases. According to a recent review, acupuncture shows therapeutic potential for more than 70 conditions, with moderate to substantial evidence supporting its efficacy in at least 8 specific diseases (2). Nonetheless, its underlying mechanisms—particularly how stimulating distal acupoints (remote from the affected area) elicits therapeutic effects through meridian pathways—remain unclear.

The focus of research on these mechanisms has undergone a paradigm shift, moving from the periphery to the central nervous system (CNS). There is growing consensus that the insertion and manipulation of acupuncture needles generate complex somatic afferent signals, which are integrated and processed within the brain (3, 4). This central neuromodulation is crucial not only for mediating analgesia—through the regulation of pain-processing regions like the anterior cingulate cortex and periaqueductal gray (5, 6)—but also for influencing higher-order cognitive and affective processes. Emerging evidence indicates that acupuncture can significantly modulate functions such as attention, emotion regulation, and interoceptive awareness by altering activity in key brain networks (7, 8).

The advancement of multi-modal neuroimaging has been instrumental in delineating these neural mechanisms, with each technique offering unique insights: functional magnetic resonance imaging (fMRI), with its high spatial resolution, excels at mapping the brain’s hemodynamic response and has been pivotal in demonstrating that acupuncture modulates functional connectivity within and between large-scale networks (e.g., DMN, SN, executive control network) in various patient populations (9, 10). Electroencephalography (EEG), providing millisecond-level temporal resolution, captures the dynamic electrophysiological brain responses to acupuncture, such as the normalization of aberrant EEG microstates in patients with post-stroke depression (11, 12). Structural MRI (sMRI) has revealed that sustained acupuncture intervention can induce neuroplastic changes, evidenced by alterations in gray matter density or white matter integrity in specific pathways (9). While these complementary approaches collectively affirm that acupuncture’s CNS effects are robust and reproducible, a direct comparison highlights a critical gap: fMRI is uniquely positioned to bridge the spatial specificity of acupoint effects with the network-level dynamics that underlie cognitive changes, making it the ideal modality for investigating how acupoint stimulation influences organized brain networks.

Despite this progress, the neuroimaging literature on acupuncture is fraught with methodological inconsistencies that obscure a clear interpretation. Many previous fMRI studies employ combinations of acupoints (13, 14), making it impossible to attribute observed effects to any single acupoint. Furthermore, studies focusing on individual acupoints are often hampered by small sample sizes (15, 16) and inadequate sham-controlled designs (16, 17), thereby conflating specific neuromodulatory effects with non-specific placebo responses.

To address these limitations, the present study employs a rigorous, single-acupoint design under a well-controlled, task-based fMRI paradigm. We focus on a therapeutically significant and commonly used acupoint to precisely investigate its distinct impact on functional brain network dynamics. Our objectives are twofold: (1) to isolate the specific central effects of a defined acupoint from non-specific responses using a robust sham-control design, and (2) to interpret these findings within the established framework of CNS network modulation, thereby contributing to a more precise understanding of acupuncture’s neural mechanisms and its potential applications in cognitive regulation.

## 2 Methods

### 2.1 Study design

**Figure 1:**
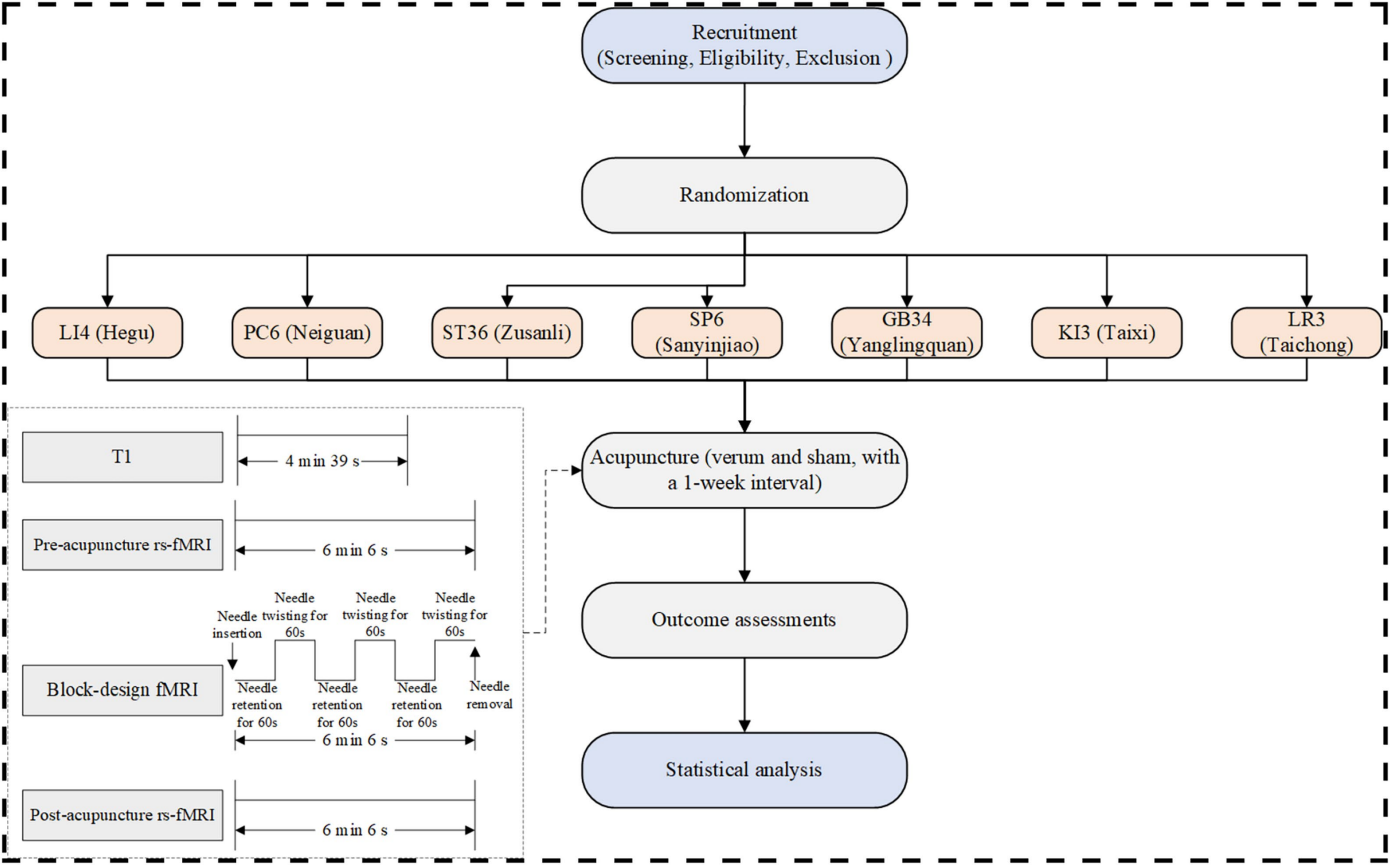
Flow chart.

**Figure 2:**
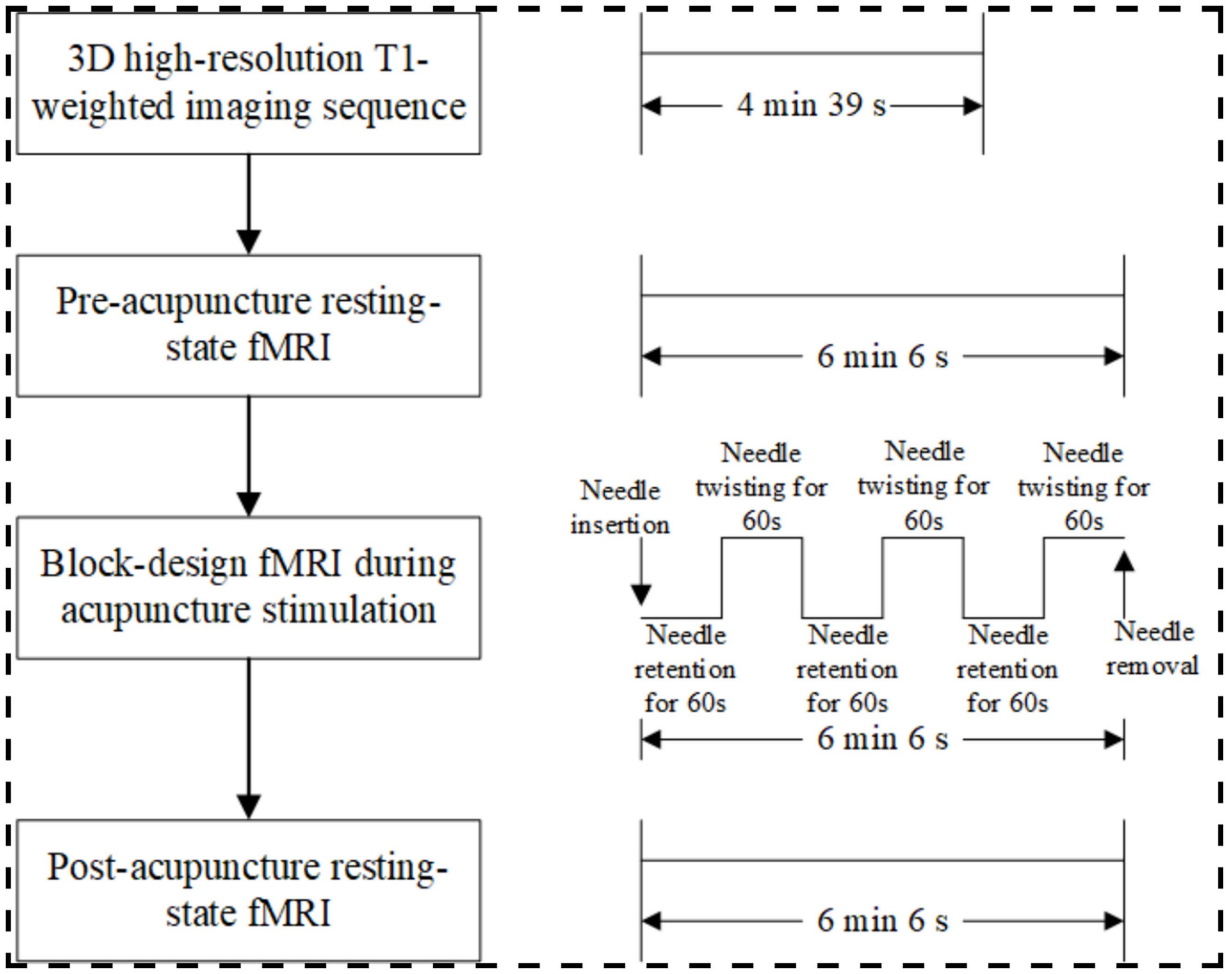
fMRI protocol.

This prospective, single-center, randomized, single-blind, placebo-controlled study will enroll 250 healthy participants. Participants will undergo acupuncture in separate sessions at one of seven predefined acupoints (verum acupuncture) and its corresponding non-acupoint (sham acupuncture), with a 1-week interval between sessions (18) (Figure 1). All participants will undergo two identical fMRI scanning sessions, separated by a 1-week interval, which corresponds to the washout period between the two acupuncture interventions. Each session will include resting-state fMRI scans before and after acupuncture, as well as task-based fMRI scans during acupuncture (Figure 2). The sequence of verum and sham protocols will be randomized across all fMRI runs, and the order of presentation will be counterbalanced across subjects (18).

### 2.2 Participants

Participants in this study will be recruited from Shenzhen Traditional Chinese Medicine Hospital. Written informed consent will be obtained from each individual prior to enrollment.

#### 2.2.1 Inclusion criteria

(1) Aged 18–45 years, regardless of gender.
(2) Right-handed.
(3) No clinically significant history of cardiovascular, respiratory, or neurological diseases.
(4) No personal or familial history of psychiatric disorders.
(5) No acupuncture treatment within the past month.
(6) No fever or use of antipyretic analgesics within the past 3 months.
(7) Willing to provide informed consent and sign the consent form.

#### 2.2.2 Exclusion criteria

(1) Pregnancy or lactation.
(2) Individuals who do not respond to acupuncture stimulation.
(3) Individuals with claustrophobia.
(4) Those with metallic implants or other contraindications for MRI.

#### 2.2.3 Withdrawal criteria during the study

(1) Participants who fail to comply with the study procedures, thereby compromising data quality.
(2) Those who experience severe adverse events or emergencies during the trial.
(3) Participants unable to complete the study as scheduled.
(4) Those exhibiting excessive head motion during MRI scanning, leading to unusable data.
(5) Participants who receive other acupuncture treatments during the trial period.

### 2.3 Sample size

Currently, there are no standardized criteria for determining sample sizes in MRI studies, as they are often constrained by practical considerations such as scanning time and cost. Based on an efficiency analysis of sample size estimation in MRI research, a previous study indicated that approximately 12 participants are required to achieve 80% power at the single-voxel level for typical activation effects (19). In line with this reference and considering the study design, we set a sample size of 30 participants per acupuncture point. Each volunteer will undergo both verum and sham acupuncture at the same acupoint, resulting in a total of 210 experimental sessions. To account for potential data loss due to head motion or participant dropout, we plan to enroll a total of 250 subjects.

### 2.4 Randomization, allocation concealment, and blinding

In this study, participants will be randomly assigned to one of the seven acupoint groups. The randomization sequence is generated using SPSS 26.0 software, and the allocation scheme is concealed using opaque sealed envelopes. Upon participant enrollment, the allocation will be revealed by sequentially opening the envelopes to determine the assigned acupoint for acupuncture. The blinding procedure involves three aspects: first, participants are blinded to the specific acupoints used in the trial; they are only informed that the acupoints are located in the distal limbs. Second, radiologists, due to their professional constraints, are unaware of the specific acupoints and their significance, ensuring the objectivity of the scanning process. Third, image processors are blinded to the participants’ group assignments, ensuring the objectivity of the image processing.

### 2.5 Intervention

**Figure 3:**
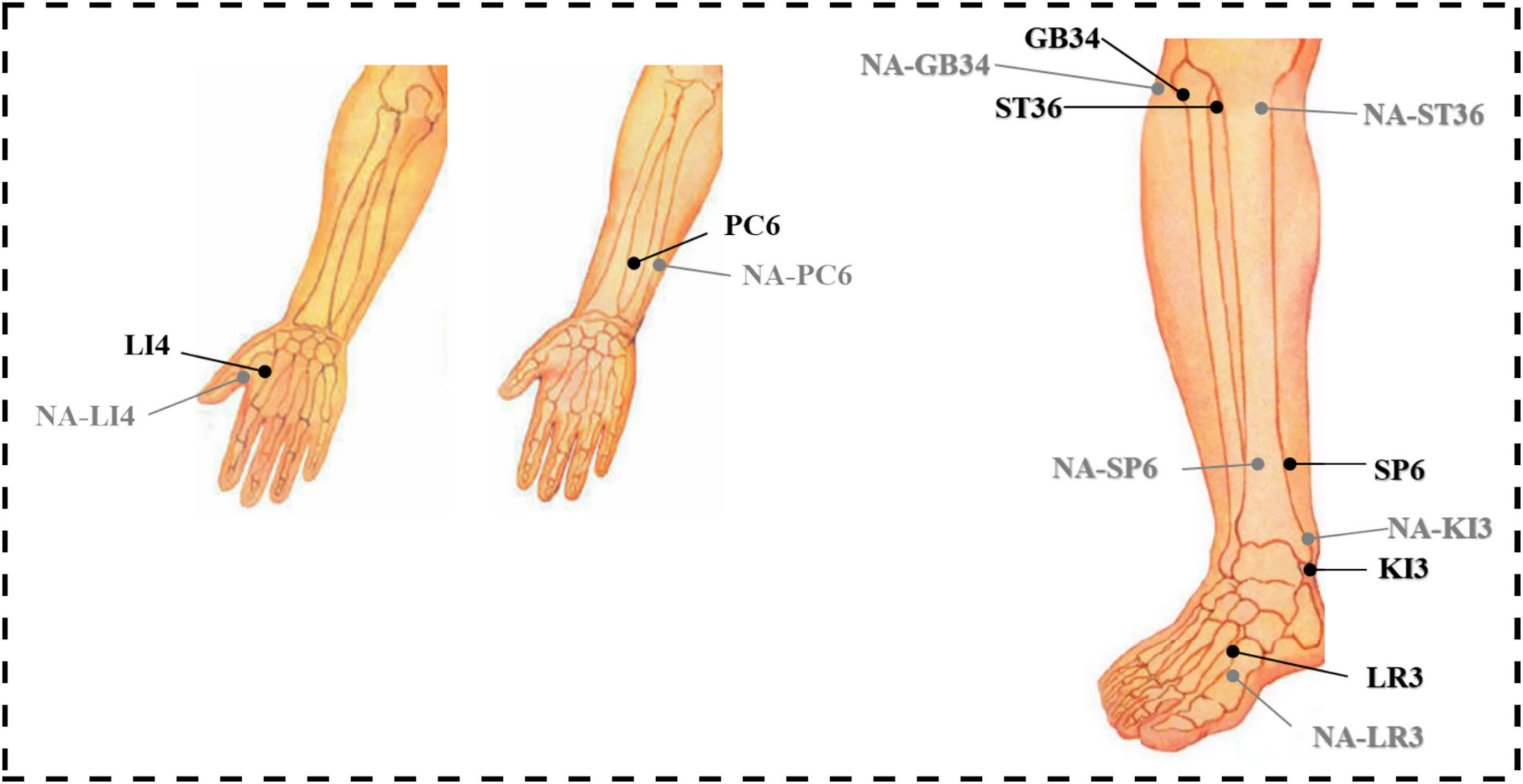
Location of acupoints and non-acupoints in the trial.

Based on a comprehensive review of fMRI studies exploring acupuncture mechanisms and clinical consensus on frequently used acupoints, this study selects the following acupoints: LI4 (Hegu), PC6 (Neiguan), ST36 (Zusanli), SP6 (Sanyinjiao), GB34 (Yanglingquan), KI3 (Taixi), and LR3 (Taichong) (Table 1 and Figure 3). These acupoints frequently employed in clinical practice to regulate the flow of Qi and blood, address imbalances in multiple organ systems (Zang-fu), and promote overall homeostasis. All acupoint localizations strictly adhere to the WHO Standard Acupuncture Point Locations in the Western Pacific Region (20).

**Table 1:** Locations of acupoints.

Acupoints	Locations	Insert depth
Hegu (LI4) (unilateral)	On the dorsum of the hand, radial to the midpoint of the second metacarpal bone	0.5–1 cun
Neiguan (PC6) (unilateral)	On the anterior aspect of the forearm, between the tendons of the palmaris longus and the flexor carpi radialis, 2 B-cun proximal to the palmar wrist crease	0.5–1 cun
Zusanli (ST36) (unilateral)	On the anterior aspect of the leg, on the line connecting ST35 with ST41, 3 B-cun inferior to ST35	1–2 cun
Sanyinjiao (SP6) (unilateral)	On the tibial aspect of the leg, posterior to the medial border of the tibia, 3 B-cun superior to the prominence of the medial malleolus	1–1.5 cun
Yanglingquan (GB34) (unilateral)	On the fibular aspect of the leg, in the depression anterior and distal to the head of the fibula	1–1.5 cun
Taixi (KI3) (unilateral)	On the posteromedial aspect of the ankle, in the depression between the prominence of the medial malleolus and the calcaneal tendon	0.5–0.8 cun
Taichong (LR3) (unilateral)	On the dorsum of the foot, between the first and second metatarsal bones, in the depression distal to the junction of the bases of the two bones, over the dorsalis pedis artery	0.5–0.8 cun

*B-cun: *proportional bone (skeletal) cun.

#### 2.5.1 Verum acupuncture group

Following skin disinfection, an opaque, non-metallic sponge pad will be positioned on the skin surrounding the target area. Non-magnetic acupuncture needles (Suzhou Acupuncture & Moxibustion Appliance Co., Ltd., China; 0.25 × 40 mm) will be inserted to 2 cm deep into the designated acupoints on the right limbs (Table 1). After Deqi (A special sensation of acupuncture characterized by pain, soreness, distension, heaviness, or numbness), twisting (90–180°, 60–90 times/min), and lifting-thrusting (0.3–0.5 cm, 60–90 times/min) were performed for 1 min. During task-based fMRI scanning, the same twisting manipulation will be performed for 60 s at 60-s intervals (synchronized with scan initiation), continuing until completion of the 6-min 6-s protocol, followed by needle removal.

#### 2.5.2 Sham acupuncture group

**Figure 4:**
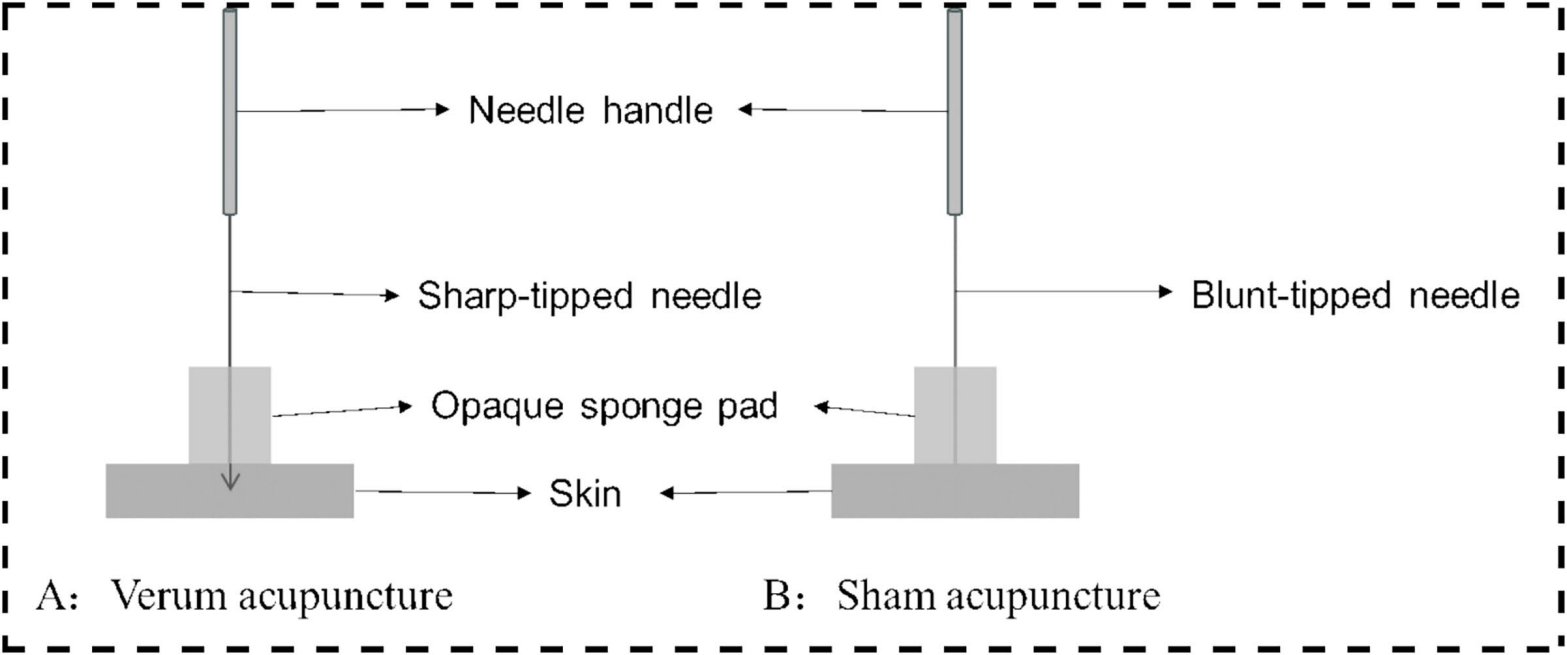
Verum and sham acupuncture.

Non-acupoints are localized at 1.5 cm lateral to the corresponding verum acupoints (Table 1), outside meridian trajectories and neurovascular bundles, avoiding tender points and hair follicles (Figure 3). Identical opaque, non-metallic sponge pads, as used in the verum group, will be placed on the skin surrounding the target area after disinfection. Blunt-tipped placebo needles (Suzhou Acupuncture & Moxibustion Appliance Co., Ltd., China; 0.25 × 30 mm) will be vertically placed on the skin surface without penetration, maintaining slight tactile pressure. Hence, the Deqi experience of the participants will not be overemphasized. Except locations of acupoints and needle penetration, the manipulation procedures and course will be the same with the verum acupuncture group (Figure 4).

#### 2.5.3 Acupuncturist qualification

To ensure consistency, licensed acupuncturist will be trained to conduct the acupuncture operations.

### 2.6 Outcome measurements

#### 2.6.1 Demographic and basic clinical information collection

The demographic information (age, gender, education, marital status and occupation) and time of enrollment will be obtained at baseline.

#### 2.6.2 MRI scanning protocol

The fMRI data will be acquired with the Siemens Prisma 3.0T MRI (Siemens, Munich, Germany) at the Shenzhen Traditional Chinese Medicine Hospital, Shenzhen, China. High-resolution T1-weighted structural images will be acquired using a magnetization-prepared rapid gradient-echo (MPRAGE) sequence with the following parameters: repetition time (TR) = 2,200 ms, echo time (TE) = 2.45 ms, field of view (FOV) = 256 mm × 256 mm, isotropic voxel resolution = 1 × 1 × 1 mm^3^, 175 slices with no interslice gap, and a flip angle (FA) of 8°. For resting-state and task-based functional scans, the parameters will be as follows: TR = 2,000 ms, TE = 30 ms, 37 slices, in-plane resolution = 64 × 64, voxel resolution = 3.75 × 3.75 × 4 mm^3^, and 240 volumes acquired per run. Before the scanning session, participants will be positioned supine with their heads immobilized using a pillow. They will be instructed to keep their eyes closed, remain relaxed, avoid deliberate mental activity, and breathe naturally. Foam earplugs are provided to minimize scanner noise. Scanning commences after participants have acclimated to the environment.

Both verum and sham acupuncture protocols will follow the procedure below (Figure 2). After 20 min of quiet rest, the subject will first undergo a 14-s locator scan to determine the imaging range. This will be followed by a 4-min and 39-s 3D high-resolution T1-weighted imaging sequence to acquire anatomical structural information. A pre-acupuncture resting-state fMRI scan lasting 6 min and 6 s will then be performed. Subsequently, a licensed acupuncturist will enter the MRI scanning room to administer acupuncture on the right limb using a block paradigm: three 60-s cycles of twisting needle manipulation will alternate with three 60-s cycles of needle-retaining baseline states. Concurrently, task-based fMRI scanning will be conducted for 6 min and 6 s. Following needle removal, a 6-min and 6-s post-acupuncture resting-state fMRI scan will be acquired.

#### 2.6.3 Blinding assessment

When recruiting and screening eligible participants, subjects will be informed that they will receive both verum and sham acupuncture stimulation while the orders of presentation remain blinded to them. Following the completion of two fMRI scanning sessions, participants will be asked to guess the type of acupuncture they received during each session to assess the blinding effectiveness. The question posed to participants will be: “Which type of acupuncture do you believe you received during the two sessions?” Participants will be provided with three response options: traditional acupuncture, sham acupuncture, or uncertain.

#### 2.6.4 Deqi sensation assessment

Following the completion of both trials, participants will be assessed for the types and intensity of Deqi sensations using the Modified Massachusetts General Hospital Acupuncture Sensation Scale-Chinese version (C-MMASS) (21). The C-MMASS evaluates 12 specific types of sensations: soreness, pain, pressure, heaviness, distension, tingling, numbness, dull ache, warmth, cold, throbbing, and other sensations. The intensity of each sensation will be rated on a scale from 0 to 10, where 0 indicates no sensation, 1–3 represents mild intensity, 4–6 moderate intensity, 7–9 severe intensity, and 10 signifies the maximum tolerable intensity.

### 2.7 Data management and statistical analysis

#### 2.7.1 Data preprocessing

The preprocessing of magnetic resonance imaging (MRI) data will involve format conversion using the dcm2niigui software, followed by subsequent preprocessing steps performed with the Statistical Parametric Mapping (SPM12) toolbox on the MATLAB R2021A platform. The workflow will include the following procedures: (1) To ensure data stability, images from the first five time points will be excluded during preprocessing. (2) All remaining scanned images will then be aligned to the middle image and corrected for head motion. (3) During motion correction, volumes exhibiting head displacement with 3D translation of >1.5 mm or 3D rotation >1.5° will be deleted (22). (4) For spatial registration, each patient’s T1-weighted images will be co-registered with their corresponding functional images. (5) The images will then be uniformly segmented and resampled. The retained data will undergo spatial normalization into Montreal Neurological Institute (MNI) space. (6) Finally, spatial smoothing will be applied using a Gaussian kernel with a full width at half maximum of 6 mm.

#### 2.7.2 Functional connectivity and network analysis

Resting-state functional connectivity (FC) analysis was employed to investigate the synchronized activity between brain regions (23, 24). Specifically, we utilized seed-based correlation analysis, a widely used method to quantify FC between specific regions of interest (25). Following standard resting-state fMRI preprocessing, the mean time series will be extracted from a predefined seed region of interest (ROI). Whole-brain connectivity maps will be generated by computing Pearson’s correlation coefficients between the seed time series and the time series of every other voxel in the brain. The resulting correlation coefficients (*r*-values) will be converted to *Z*-scores using Fisher’s *Z*-transformation to normalize their distribution for subsequent group-level statistical analysis.

The resulting connectivity matrices were then used to construct brain networks where nodes were defined according to the Dosenbach-160 atlas (26), and edges represented the significant FC between node pairs. Subsequently, graph-theoretical analysis was performed to characterize the topological properties of these networks using measures such as clustering coefficient and characteristic path length (27, 28). All analyses were carried out using the GRETNA toolbox (29). Using these nodes and the Fisher’s *Z*-transformed connectivity strengths as weighted edges, we will construct individual functional brain networks for each subject and time point. Key graph-theoretic metrics, including global efficiency (quantifying global integration), local efficiency (quantifying local specialization and fault tolerance), and nodal degree/strength (quantifying regional influence), will be computed from these networks across a range of connection densities to characterize their topological organization robustly.

#### 2.7.3 Statistical analysis

Neuroimaging analyses will be conducted using the SPM12 toolbox on the MATLAB platform. First, one-sample *t*-tests will be performed to identify brain activation patterns associated with verum acupuncture (at true acupoints) and sham acupuncture (at non-acupoints). Subsequently, repeated-measures analysis of variance (ANOVA) will be applied. If statistically significant effects are detected, paired-sample t-tests will be conducted between the two groups to identify differential brain activation patterns between true acupoints and non-acupoints. Additionally, paired-sample *t*-tests will be performed to compare: (1) Resting-state functional activity pre- versus post-acupuncture; (2) Resting-state activity (pre-acupuncture) versus task-based activity (during acupuncture). A voxel-wise threshold of *p* < 0.005 with a false discovery rate-corrected cluster level of *p* < 0.05 was applied in the task fMRI analysis. Significant activation clusters will be visualized and localized using the xjView, MRIcron, and BrainNet Viewer toolboxes within the MATLAB platform.

#### 2.7.4 Common brain effects of acupoints, pain-related brain regions, and differential brain effects

Analyses of common brain effects elicited by acupoint stimulation will be performed using GingerALE software (Version 3.0.2, http://brainmap.org/ale) with cluster-level family-wise error (FWE) correction. The following parameters will be applied: 5,000 permutations for statistical testing, a cluster-forming threshold of *p* < 0.001, and a statistical significance threshold of *p* < 0.05 (cluster-level FWE corrected) (30). Additionally, a predefined pain-related brain map (derived from the Neurosynth database, https://neurosynth.org) will be used to evaluate spatial overlap between acupoint-induced activation clusters and pain-associated brain regions. The overlap analysis will adopt a statistical significance threshold of *p* < 0.05.

#### 2.7.5 Functional decoding

To determine the functional roles of acupoint-induced activation clusters, forward inference and reverse inference analyses will be conducted using the BrainMap database.^1^ Forward inference will decode the behavioral domains (BD) and paradigm classes (PC) associated with the identified brain regions. Behavioral domains are hierarchically categorized into five major classes (*behavioral, cognitive, emotional, interoceptive, and perceptual*) and 51 subclasses. For forward inference, the functional profile of a brain region will be defined by the probability of its involvement in specific behavioral or cognitive tasks based on task-classification labels. Reverse inference will identify the most likely behavioral domains and paradigm classes linked to the functional connectivity patterns of activation clusters using Bayesian probability.^2^ This analysis will quantify the posterior probability that a specific brain region is engaged during a particular task or behavioral context.

The significance of forward and reverse inference results will be assessed using likelihood ratio tests and chi-square tests, respectively. A false discovery rate (FDR) correction for multiple comparisons will be applied to both analyses, with a significance threshold of *p* < 0.05.

#### 2.7.6 Statistical analysis of baseline information and C-MMASS

Statistical analyses of participants’ baseline characteristics and inter-group comparisons of C-MMASS scores will be performed using SPSS software (version 26.0). Categorical variables between groups will be analyzed with the chi-square test, while continuous variables will be assessed using independent samples *t*-tests. Within-group comparisons of categorical variables (pre- vs. post-intervention) will employ the chi-square test, and continuous variables will be evaluated with paired samples *t*-tests. All quantitative data will be presented as mean ± standard deviation. A threshold of *p* > 0.05 will indicate no statistically significant difference.

### 2.8 Safety measurements

All adverse events must be documented in detail in the Case Report Form (CRF), including symptom characteristics, severity, onset/duration, management measures, and outcomes. An objective assessment of the causality between the event and the acupuncture intervention is required. The attending physician may discontinue a participant’s involvement based on clinical judgment; such decisions require the signature and date from the researcher.

Regarding MRI examinations, participants may experience discomfort from the prolonged scanning time and acoustic noise. Headphones will be provided to reduce noise exposure. If a participant remains unable to tolerate the procedure, the scan may be terminated by mutual agreement, and this will be recorded as a study withdrawal. The decision to resume scanning after a rest period will be based on the physician’s assessment of the participant’s readiness.

## 3 Results

### 3.1 Immediate brain responses during task fMRI

#### 3.1.1 Common brain effects

Both verum and sham acupuncture may commonly activate a brain network involving the anterior cingulate cortex, anterior insula, primary/secondary somatosensory cortices, and prefrontal cortex. This network is responsible for processing stimulus salience, bodily sensations, and cognitive appraisal, reflecting the non-specific effects of acupuncture. That is, this brain activity largely stems from shared components such as attention, sensation, and expectation elicited by the skin penetration itself, overlapping with the neural substrates of placebo effects.

#### 3.1.2 Differential brain effects

Verum acupuncture is hypothesized to produce more robust and widespread brain modulation than sham acupuncture, engaging distinct neural mechanisms. Specifically, it activates subcortical centers (e.g., hypothalamus, brainstem) and limbic regions (e.g., amygdala, hippocampus), implicating its role in autonomic, affective, and homeostatic regulation. In parallel, it more strongly suppresses the core default mode network (DMN), including the posterior cingulate and medial prefrontal cortices. This pronounced DMN deactivation may reflect an enhanced redirection of attention toward bodily stimuli, aligning with the concept of “regulating the spirit” and distinguishing the holistic action of verum acupuncture.

### 3.2 Resting-state fMRI: functional connectivity and network analysis

This aspect may yield the most intriguing findings, as it reflects the sustained after-effects of acupuncture, rather than immediate responses.

#### 3.2.1 Pre- vs. post-acupuncture changes

Following verum acupuncture, significant reorganization of resting-state functional connectivity occurs compared to sham acupuncture. This includes weakened connectivity within the default mode network (DMN), suggesting that the brain may remain less prone to mind-wandering even at rest; enhanced coupling between the salience network (SN) and the central executive network (CEN), indicating a potential improvement in the brain’s ability to flexibly switch between internal Deqi sensation and external tasks; and reduced hyper-connectivity among pain-related regions such as S1, S2, thalamus, and anterior insula, which may underlie its long-term analgesic effects.

#### 3.2.2 Graph theory network metrics

Verum acupuncture may promote a more optimal topological organization of the brain’s functional network.

### 3.3 Correlation between subjective sensations (Deqi—C-MMASS) and objective brain activity

The intensity and multidimensional characteristics of the “Deqi” sensation will correlate strongly with specific patterns of brain activity. The composite intensity of sensations like soreness, numbness, distension, and heaviness may positively correlate with activation intensity in the insula and ACC.

### 3.4 Functional decoding analysis

Decoding via databases like BrainMap will reveal that the brain clusters activated by verum acupuncture are associated with functional domains far beyond mere “sensory processing.” major behavioral domains (BD) and paradigm classes (PC) include areas such as sensation, perception, and interoception.

### 3.5 Blinding assessment and placebo effects

The blinding assessment may show that a portion of participants cannot accurately distinguish between verum and sham acupuncture, with a potentially high proportion selecting “uncertain.”

If the subjective guesses between groups do not differ significantly, then the differences in brain responses are more likely attributable to the biological effects of acupuncture itself rather than participant expectation. This would enhance the credibility of the findings. Even if placebo effects are present, this study is well-designed to dissociate them (common effects) from the specific effects (differential effects) using neuroimaging.

## 4 Discussion

In this study, we will examine whether acupoints engage both shared and distinct brain regions during neural regulation, with the specificity of these regions potentially underlying their unique therapeutic functions.

Our aim is to establish a bridge between traditional medicine and modern medicine through this research. Based on traditional Chinese medicine theory, LI4 (Hegu) is primarily used for pain relief and disorders of the head and face, with strong regulatory effects on Qi and blood. PC6 (Neiguan) harmonizes the stomach, alleviates nausea and vomiting, calms the Shen, and addresses cardiac and chest discomfort. ST36 (Zusanli) tonifies the whole body, strengthens the spleen and stomach, improves digestion and energy, and supports immunity. SP6 (Sanyinjiao), where the spleen, liver, and kidney meridians converge, is key in treating gynecological and urogenital disorders and nourishing blood and Yin. GB34 (Yanglingquan), the influential point for tendons, is essential for musculoskeletal conditions and lateral body pain. KI3 (Taixi), the kidney source point, tonifies kidney essence (Jing) and addresses deficiencies in both kidney Yin and Yang, which are foundational in aging and chronic disease. LR3 (Taichong), the liver source point, soothes the liver, promotes Qi flow, alleviates stress and emotional constraint, and treats disorders related to liver Qi stagnation. For instance, we anticipate that acupuncture at ST36 will significantly modulate the DMN and the salience network. Notably, DMN activity is closely linked to self-referential processing, introspection, and impairments of the gut-brain axis in gastrointestinal diseases (31, 32). Thus, we expect that our results may provide a neuroscientific basis for the action of ST36: by regulating the DMN and visceral sensory regions, ST36 could help restore homeostasis of the gut-brain axis, offering a modern biological correlate to its traditional role in “harmonizing the stomach and spleen.”

In terms of the mechanism of fMRI, previous review demonstrated that GB34, an acupoint mainly used in stroke and Parkinson’s disease, could activate brain response in the premotor cortex, the supplementary motor area, and the supramarginal gyrus (33). And Liu et al. (34) found that there is signal synchronization change in ReHo in different brain regions including cognitive, motor, default network, limbic system and other parts of the encephalic region following acupuncture at GB34. Our meta-analysis also found that ST36 could activate the left cerebellum, the bilateral rolandic operculum, the right supramarginal gyrus, and the right cerebellum, which are mainly associated with action and perception (35). Acupuncture at PC6 could provoke extensive signal attenuations in the cerebrocerebellar and subcortical area, and selectively evoke neural responses of the insula, hypothalamus, and flocculonodular lobe of cerebellum (nodulus and uvula) (36). The limbic/paralimbic-cerebellum and subcortical areas showed extensive causal interactions following acupuncture at PC6 (37). Acupuncture at SP6 could activate the default mode network, descending pain modulation pathway and visual cortices. He et al. (38) found that the decreased ReHo after acupuncture at KI3 was concentrated in the left postcentral, right paracentral lobule, and right SMA, which are important for processing sensory information and motor control. Furthermore, acupuncture at KI3 has a specific effect on certain brain regions associated with perception, body movement, spirit, and association (22). The latest meta-analysis (39) confirmed that acupuncture at the LR3 could activate regions such as the right postcentral gyrus, left thalamus, left middle frontal gyrus, and right superior frontal gyrus. Those activated regions align with the basal ganglia network, auditory network, left executive control network, posterior salience network, right executive control network, and sensorimotor networks, which are related to pain perception, emotional processing, and linguistic functions. While our analysis of fMRI activation clusters evoked by individual acupoints may demonstrate spatial convergence with those existing reports, this study will advance the field by decoding the functional significance of these regions. Specifically, we will employ forward and reverse inference approaches to elucidate whether the observed activations map onto established neural networks—transcending purely descriptive localization toward mechanistic interpretation.

This study is designed to have substantial translational potential. First, the single-acupoint fMRI paradigm we establish will serve as a standardized control for future research, aiding in the identification of acupoint-specific neural signatures. For example, comparing cerebral responses between ST36 (which strengthens the spleen) and LV3 (Taichong, which soothes the liver) may reveal differences in brain network representations of TCM functional systems corresponding to the liver and spleen. Second, specific brain network response patterns—such as the extent of DMN suppression—will be evaluated as potential objective biomarkers for predicting treatment response, thereby advancing acupuncture toward “precision medicine.” Future studies will recruit clinical populations (e.g., patients with irritable bowel syndrome) to undergo fMRI before and after treatment, with the aim of validating these biomarkers and exploring underlying molecular mechanisms.

The selection of healthy participants is primarily motivated by the need to eliminate confounding effects arising from underlying pathological states on brain functional responses. To begin with, patients, especially those with neurological disorders, often exhibit abnormal regional brain activation, brain network dysfunction and abnormal functional connectivity within the brain (40–43). Consequently, fMRI signal changes observed in patient cohorts become ambiguous, making it challenging to definitively attribute those alterations to the acupuncture intervention itself or the inherent neuropathological features of the underlying disease. Secondly, the responses to acupuncture in patients may differ from those in healthy individuals (44–46). Therefore, in this trial, we will recruit healthy subjects rather than patients.

Currently, there are two major paradigms for task-based fMRI study: non-repeated event-related (NRER) design and block design. NRER designs effectively capture transient neural responses to discrete stimuli (e.g., instantaneous needle insertion) (47). In contrast, our block design paradigm better reflects real-world clinical acupuncture practice which comprises four sequential phases: needle insertion, needle manipulations (lifting-thrusting and rotating) to elicit Deqi, needle retention and needle withdrawal. Acupuncture elicits prolonged neural and hemodynamic responses rather than transient bursts of activity. Block design, with its alternating periods of stimulation and rest (e.g., 60 s needle manipulation followed by 60 s rest), optimally captures these sustained effects. Furthermore, as the most commonly applied and the most efficient paradigm (46, 47), block design has the advantages of a relatively large blood oxygen level dependent (BOLD) signal change relative to baseline (48), superior signal-to-noise ratio (SNR) and increased statistical power (47, 49).

However, there are some limitations in this study. Firstly, due to resource constraints, this study will be unable to encompass all acupoints. Future work will be needed to construct a comprehensive brain mapping encompassing all acupoints to better inform clinical practice. Secondly, considering the characteristics of acupuncture, it is difficult to design a practitioner-blinded trial. Thirdly, the study will be limited to manual acupuncture, which may introduce variability due to subtle differences in needle manipulation techniques. Furthermore, different acupuncture modalities (e.g., electroacupuncture vs. manual acupuncture) may induce different effects (46, 50, 51). Future studies should compare outcomes across different acupuncture modalities to determine modality-specific neural correlates. Finally, although a sham acupuncture control will be employed, this approach may not eliminate the influence of placebo effects inherent in acupuncture trials.

## Footnotes

^1^ http://www.brainmap.org

^2^ http://brainmap.org/scribe/
